# Massive Neurocysticercosis: Encephalitic versus Non-encephalitic

**DOI:** 10.4269/ajtmh.2012.12-0162

**Published:** 2012-09-05

**Authors:** Oscar H. Del Brutto, Xavier Campos

**Affiliations:** Department of Neurological Sciences, Hospital, Clinica Kennedy, Guayaquil, Ecuador; Outpatient Center North, Ecuadorian Institute of Social Security, Guayaquil, Ecuador

Most patients with neurocysticercosis have few intracranial lesions. However, a small subset show development of massive infections that may be divided into encephalitic and non-encephalitic. Proper differentiation of both forms is important because they have different pathogenetic mechanisms and require different therapeutic approaches.

**Figure 1.**
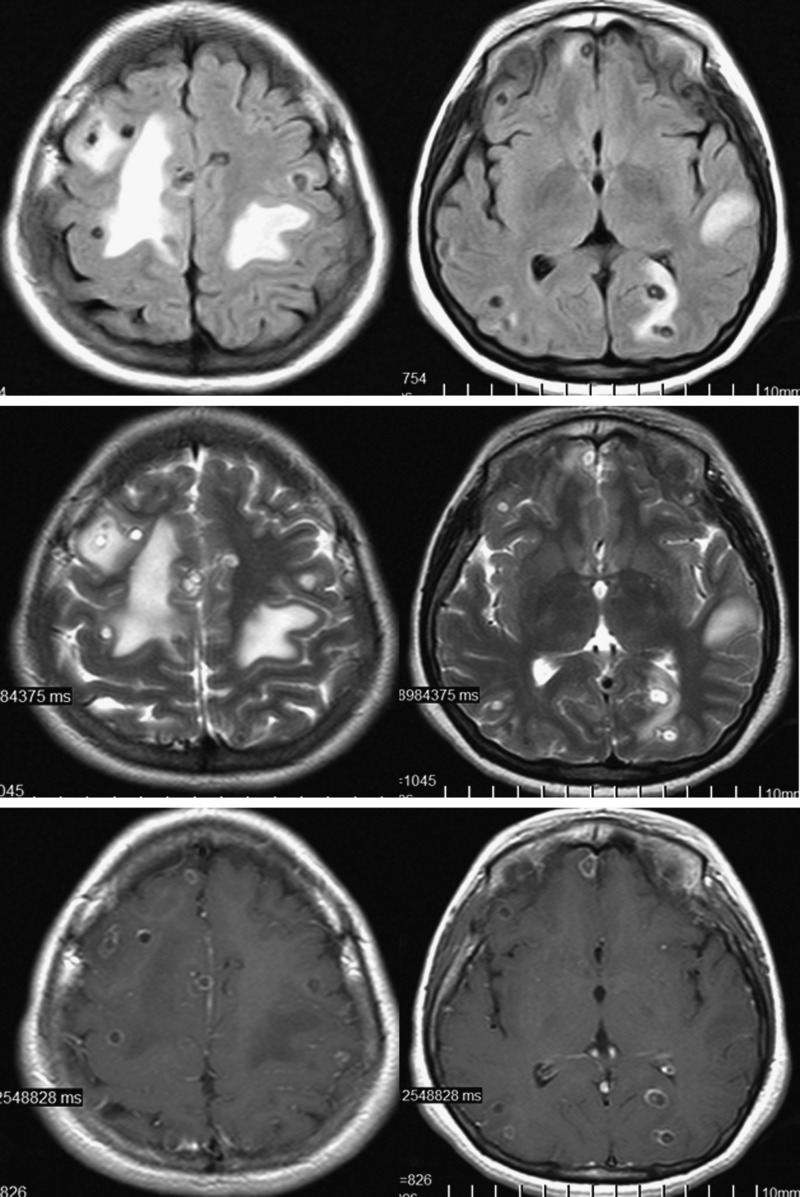
Magnetic resonance imaging of patient with encephalitic neurocysticercosis, showing multiple degenerating cysticerci and marked brain swelling.

The encephalitic form often occurs in children and young women who have not had contact with the parasite until they are infected with a heavy load of *Taenia solium* eggs.^1^ In these cases, the host's immune system actively reacts against the parasites. The clinical picture is that of acute encephalitis, and neuroimaging shows multiple degenerating cysticerci and marked brain swelling (Figure 1). Cysticidal drugs are not needed because most parasites will die spontaneously. Moreover, use of these drugs can exacerbate the inflammatory reaction and be harmful to patients.

**Figure 2.**
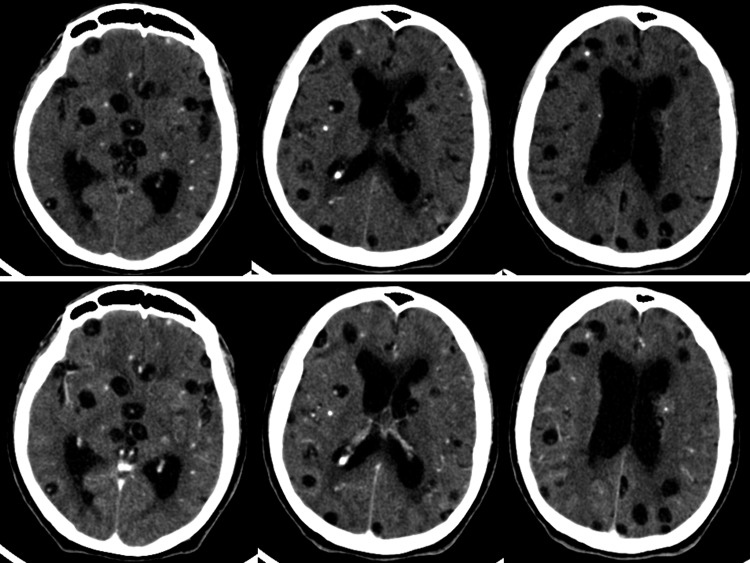
Computed tomography of patient with heavy non-encephalitic neurocysticercosis, showing multiple viable cysticerci and no edema.

In contrast, heavy non-encephalitic neurocysticercosis occurs most often in *T. solium* carriers who have shown development of mechanisms of immune tolerance to nervous system invasion by cysticerci and in patients with chronic seizure disorders and normal results for neurologic examinations.^2^ Neuroimaging shows multiple viable cysticerci and no edema (Figure 2). These patients may benefit from cysticidal drug therapy, and some need repeated courses of therapy to overcome the infection. Also, patients must be treated if they are found to be carriers of *T. solium*.
